# Comparison of Single‐Coil Versus Dual‐Coil Implantable Cardioverter Defibrillator Devices: A Systematic Review and Meta‐Analysis of Efficacy and Extraction‐Related Outcomes

**DOI:** 10.1002/clc.70083

**Published:** 2025-02-05

**Authors:** Muhammad Fawad Tahir, Adeena Maryyum, Zainab Mubbashir, Abdul Moiz Khan, Syed Irtiza Imam, Fatima Mustafa, Syeda Zahra Hasan, Umer Shoaib, Areej Iqbal, Osama Saeed, Manisha Purushotham, Maimoona Khan, Shahtaj Tariq, Muhammad Omar Larik, Muhammad Umair Anjum, Muhammad Hasanain, Tanesh Ayyalu, Mah I. Kan Changez, Javed Iqbal

**Affiliations:** ^1^ Department of Medicine H. B. S. Medical and Dental College Islamabad Pakistan; ^2^ Department of Cardiology Rawalpindi Institute of Cardiology Rawalpindi Pakistan; ^3^ Department of Medicine Ayub Medical College Abbottabad Pakistan; ^4^ Department of Pharmacology University of Karachi Karachi Pakistan; ^5^ Department of Medicine Jinnah Sindh Medical University Karachi Pakistan; ^6^ Department of Medicine Ziauddin University Karachi Pakistan; ^7^ Department of Medicine Dow Medical College Karachi Pakistan; ^8^ Department of Medicine Frontier Medical College Abbottabad Pakistan; ^9^ Department of Medicine Kasturba Medical College Manipal India; ^10^ Department of Medicine Dow International Medical College Karachi Pakistan; ^11^ Department of Cardiology MedStar Georgetown University Hospital Washington DC USA; ^12^ Department of Cardiac Surgery Yale University New Haven Connecticut USA; ^13^ Nursing Department Hamad Medical Corporation Doha Qatar

**Keywords:** dual‐coil, ICD, implantable cardioverter defibrillators, lead extraction, single‐coil

## Abstract

**Background:**

Implantable cardioverter defibrillators (ICD) are battery‐operated devices used to manage irregular heart rhythms and deliver therapeutic shocks to the heart. This updated systematic review and meta‐analysis compares the efficacy and extraction‐related outcomes of single‐coil versus dual‐coil ICDs in view of conflicting data.

**Methods:**

Several databases, including PubMed, Cochrane Library, and Google Scholar, were comprehensively explored dating from inception to April 1, 2024. Data were compared using odds ratio (OR), hazard ratio (HR), and mean differences (MD). A value of *p* < 0.05 indicated statistical significance.

**Results:**

Ultimately, 28 studies were included in this quantitative synthesis. Defibrillation threshold (DFT) indicated statistical superiority in the dual‐coil cohort (MD: 0.58; 95% confidence interval [CI]: 0.07–1.09; *p* = 0.03). In addition, all‐cause mortality was significantly elevated in the dual‐coil cohort (HR: 0.91; 95% CI: 0.87–0.97; *p* = 0.001). Furthermore, implant time was significantly lower in the single‐coil group (MD: −7.44; 95% CI: −13.44 to −1.43; *p* = 0.02). Other outcomes, including first shock efficacy, cardiac mortality, post‐extraction major complications, post‐extraction procedural success, and post‐extraction mortality, did not demonstrate any significant statistical differences.

**Conclusion:**

In conclusion, despite the desirable safety profile of single‐coil ICDs, the use of dual‐coil ICDs continues to hold merit due to their superior efficacy and advanced sensing capabilities, especially in complex cases. In addition, the perceived risk of a greater adverse profile in dual‐coil lead extraction can be refuted by preliminary aggregate results generated within this meta‐analysis. However, further robust studies are warranted before arriving at a valid conclusion.

## Graphical abstract

A comprehensive meta‐analysis of 28 studies identified the superiority of dual‐coil devices in terms of defibrillation threshold. In contrast, the single‐coil devices exhibited a decreased rate of mortality, in addition to a lower implant time. In conclusion, despite the desirable safety profile of single‐coil ICDs, the use of dual‐coil ICDs continues to hold merit due to their superior efficacy and advanced sensing capabilities, especially in complex cases. Further robust studies are warranted before arriving at a valid conclusion.

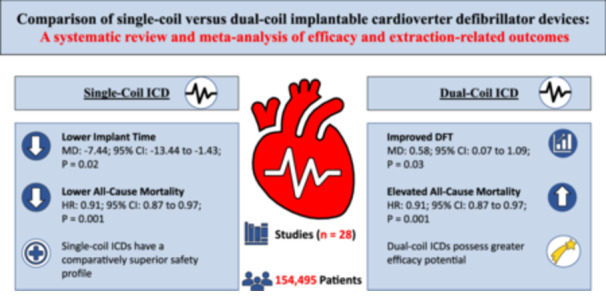

## 1 Introduction

Implantable cardioverter defibrillator (ICD) is a battery‐operated device used to manage irregular heart rhythms and deliver therapeutic shocks to the heart once detected [1]. ICDs are currently indicated for primary prevention of fatal arrhythmia, with a heightened risk of recurrence [2, 3], in addition to secondary prevention in individuals with arrhythmogenic right ventricular cardiomyopathy who have experienced sudden cardiac death [4]. Additionally, backup bradycardia pacing and heart rhythm monitoring with electrogram storage are further features this device can demonstrate [5].

Since the 1980s, implanting ICDs has become a common practice amongst healthcare providers [6]. The adoption of ICD implantation for preventing sudden cardiac death in patients with left ventricular dysfunction gained widespread acceptance following the publication of the Multicenter Automatic Defibrillator Implantation Trial II (MADIT II) study in 2002 and the Sudden Cardiac Death in Heart Failure Trial (SCD‐HeFT) study in 2005 [7, 8].

An important innovation, for optimizing patient outcomes, is the opportunity of implanting either a single‐coil or dual‐coil lead system. In a single‐coil ICD, one defibrillator lead is implanted in the right ventricle, whereas a dual‐coil ICD features a right atrial pacing lead and a right ventricular defibrillator lead [5]. While single‐coil leads are usually used with simpler implantation techniques and potentially lower procedural complications, dual‐coil leads are usually favored for their increased defibrillation efficacy and reduced risk of lead failure [9].

This systematic review and meta‐analysis aims to provide a comprehensive review of published evidence on single‐coil versus dual‐coil ICD systems. In view of conflicting data presently available, and the perceived greater adverse profile of dual‐coil ICDs warranted further evaluation into this comparison. Ultimately, this creates an indispensable tool for clinicians, researchers, and policymakers to create an evidence‐based recommendation of ICDs in light of their efficacy and safety data.

## 2 Methodology

### 2.1 Search Strategy and Databases

A comprehensive electronic search was conducted across three databases: PubMed, Cochrane Library, and Google Scholar, in accordance with the Preferred Reporting Items for Systematic Review and Meta‐Analysis Statement (PRISMA, 2020), covering articles published from inception to April 1, 2024. The search aimed to identify studies involving human subjects that compared the outcomes of single‐coil and dual‐coil ICD leads. No language restrictions or filters were applied during the search. The complete search strategy is available in Table S1.

### 2.2 Study Selection and Eligibility Criteria

Studies retrieved from the database search were subject to deduplication via EndNote Reference Library (Version X7.5; Clarivate Analytics, Philadelphia, Pennsylvania). Subsequently, an exhaustive screening process was completed through a title‐and‐abstract level search, followed by a detailed full‐text review of each relevant study. The screening process was conducted by two investigators (A. M. and A. M. K.), with any discrepancies resolved by a thorough evaluation via a third investigator (M. F. T.).

To capture a comprehensive understanding of the topic, a range of study designs were included. Specifically, observational studies, randomized controlled trials (RCTs), as well as both prospective and retrospective nonrandomized observational studies involving adult populations were considered for inclusion in order to maximize the robustness of the quantitative synthesis. Additionally, all studies reporting a direct comparison of single‐coil versus dual‐coil results were eligible for inclusion, conditional to reporting at least one relevant outcome to our analysis.

Studies were excluded via the screening process in cases of: (i) insufficient data or abstract‐only publication, (ii) single‐arm results, and (iii) the use of nonstandard current configurations (e.g., those involving leads in the inferior vena cava, coronary sinus, or azygos vein). Studies in the form of letters, case reports, and systematic/narrative reviews were not considered.

### 2.3 Data Extraction and Outcomes

The following outcomes were considered for analysis, including: (i) defibrillation threshold (DFT), (ii) first shock efficacy (FSE), (iii) all‐cause mortality, (iv) cardiovascular mortality, (v) implant time, (vi) major complications during extraction, (vii) procedural success during extraction, and (viii) extraction‐related mortality.

Additionally, the following baseline parameters were extracted, including: (i) total number of patients, (ii) follow‐up time duration, (iii) data source, (iv) study design, (v) device type, and (vi) coronary artery disease prevalence.

### 2.4 Risk of Bias Assessment

The Cochrane Risk of Bias Tool for Randomized Controlled Trials [10], Newcastle–Ottawa Scale for Cohort Studies [11], and Risk of Bias in Non‐randomized Studies of Interventions (ROBINS‐I) were utilized to evaluate the quality and identify the risk of biases present within included studies. The evaluation process was initiated by two independent reviewers (S. Z. H. and S. I. I.), with an independent third reviewer (Z. M.) available to resolve any discrepancies through mutual discussion.

### 2.5 Statistical Analysis

Data analysis was conducted using Review Manager 5.4 (Copenhagen: The Nordic Cochrane Centre, The Cochrane Collaboration, 2020). Summary estimates of univariate risk were computed using the random‐effects model, based on Mantel–Haenszel or generic inverse‐variance meta‐analytic statistical approach. The odds ratio (OR) or hazard ratio (HR) were utilized for dichotomous outcomes, whereas mean differences (MD) were employed for continuous outcomes. The 95% confidence interval (CI) values were computed for all outcomes. Heterogeneity was considered significant when *I* ^2^ > 50% [12]. A *p* < 0.05 was considered statistically significant throughout the analysis.

Prespecified subgroup analyses were performed based on: (i) study design—segregated based on RCT, nonrandomized controlled trial (N‐RCT), prospective observational, and retrospective observational approaches, and (ii) study population—segregated based on greater/less than 100 patients. Sensitivity analysis using the leave‐one‐out method was employed for outcomes demonstrating significant (*I* ^2^ > 50%) levels of heterogeneity.

## 3 Results

### 3.1 Identification of Studies and Categorization

The initial publication search yielded 1771 results. After removing 694 duplicates, 1077 studies were screened based on the abstract and title of the studies, and 655 were excluded. In the next step, we conducted a full‐text review of 422 studies and excluded the ones that did not meet our eligibility criteria. Twenty‐eight studies met our criteria and were included in the meta‐analysis. Subgroup analyses, wherever appropriate, were performed on the basis of study design (i.e., RCT, N‐RCT, prospective observational, and retrospective observational approaches), or study population (population greater/less than 100 patients). The complete PRISMA flowchart is available in Figure S1.

### 3.2 Study Characteristics and Quality Assessment

We included 28 studies in our meta‐analysis: 9 were RCTs, 15 were cohort studies, and 4 were N‐RCT. There was a higher number of male patients than females. Comprehensive information regarding patient baseline study and clinical characteristics is available in Table S2.

According to the risk of bias assessment conducted for cohort studies via the Newcastle–Ottawa scale, most studies showed a low risk of bias (Table S3) with some being at moderate risk. However, evaluation of RCTs using the Cochrane Risk of Bias tool demonstrated a mixture of moderate‐to‐high risk of bias (Figure S2) as most RCTs lacked sufficient data regarding random sequence generation and allocation concealment within their respective methodology sections. Similarly, N‐RCTs were also at a moderate‐to‐high risk of bias (Table S4).

### 3.3 Defibrillation Threshold Testing (DFT)

**Figure 1:**
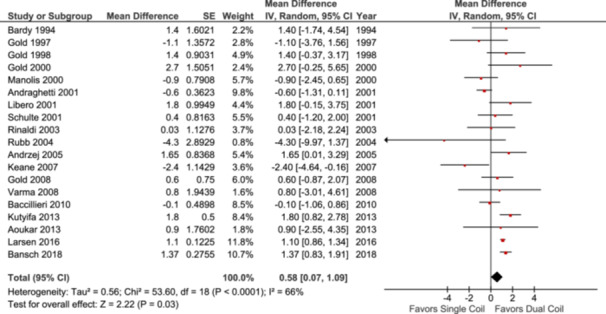
Forest plot comparing defibrillation threshold (DFT).

Nineteen studies were eligible for analysis of DFT, with a total of 10 445 patients. The analysis showed that DFTs were lower in the dual‐coil arm than the single‐coil ICDs (MD: 0.58; 95% CI: [0.07–1.09]; *p* = 0.03; Figure 1). However, substantial heterogeneity (*p* < 0.0001, *I* ^2^ = 66%) was reported for these results. To address this, we performed a sensitivity analysis using the leave‐one‐out method, but remained unsuccessful in eradicating heterogeneity.

Subgroup analyses were performed based on study design and number of patients. The analysis done on RCTs showed a statistically significant difference between the single versus dual‐coil arm, favoring the dual‐coil arm (MD: 1.08; 95% CI: [0.48–1.69]; *p* < 0.0004; Figure S3), whereas no significant differences were observed within N‐RCTs (MD: 0.23; 95% CI: [−1.30 to 1.77]; *p* = 0.77; Figure S3). The analysis of prospective studies showed insignificant results (MD: 0.15; 95% CI: [−1.50 to 1.81]; *p* = 0.86; Figure S3). Similarly, the retrospective studies showed no significant difference between the two arms (MD: 0.59; 95% CI: [−0.57 to 1.75]; *p* = 0.32; Figure S3). The studies with patients more than 100 had a statistically significant result favoring dual‐coil implants (MD: 0.80; 95% CI: [0.20–1.40]; *p* = 0.009; Figure S4). The group with < 100 patients showed no significant differences (MD: 0.20; 95% CI: [−0.75 to 1.15]; *p* = 0.68; Figure S4).

### 3.4 All‐Cause Mortality

**Figure 2:**
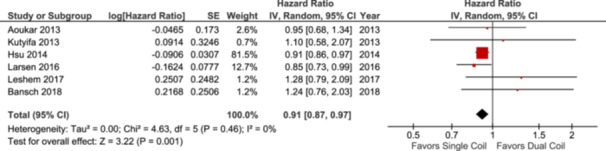
Forest plot comparing all‐cause mortality.

Data for all‐cause mortality were reported in six studies. The mortality data were available on 140 621 patients. The results for all‐cause mortality indicated lower mortality rates in the single‐coil arm compared to the dual‐coil ICD leads (HR: 0.91; 95% CI: [0.87–0.97]; *p* = 0.001; Figure 2). Considering subgroup analysis for the type of studies, the data from the prospective studies showed no statistically significant difference in all‐cause mortality in single‐coil compared to dual‐coil ICDs (HR: 0.99, 95% CI: [0.75–1.32]; *p* = 0.95; Figure S5). A single study reporting RCT did not reveal any difference between the single versus dual‐coil arm (HR: 1.24; 95% CI: [0.76–2.03]; *p* = 0.39; Figure S5). Two studies reporting N‐RCTs showed no statistically significant difference between single versus dual coil arm (HR: 0.98; 95% CI: [0.73–1.33]; *p* = 0.92; Figure S5).

### 3.5 First Shock Efficacy

**Figure 3:**
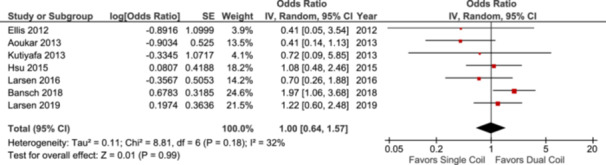
Forest plot of first shock efficacy.

Seven studies reported FSE, encompassing data on 2713 patients. When these studies were analyzed, there was no statistically significant difference in FSE between patients with single‐coil ICDs and those with dual‐coil ICDs (OR: 1.00; 95% CI: [0.64–1.57]; *p* = 0.99; Figure 3).

A further subgroup analysis was conducted based on the study design. The analysis of two RCTs showed a statistically significant in FSE with dual‐coil ICD leads compared to single‐coil leads (OR: 1.60; 95% CI: [1.00–2.56]; *p* = 0.05; Figure S6). The two N‐RCT studies had no significant difference in FSE in single‐coil ICD leads compared to dual‐coil leads (OR: 0.45; 95% CI: [0.18–1.14]; *p* = 0.09; Figure S6). When the two retrospective studies were analyzed, they also showed no significant difference in FSE with single‐coil ICD leads compared to dual‐coil leads (OR: 0.64; 95% CI: [0.26–1.57]; *p* = 0.33; Figure S6). The results were similar for prospective observational studies (OR: 1.08; 95% CI: [0.48–2.46]; *p* = 0.85; Figure S6). The subgroup analysis of the studies involving more than 100 patients, which included 6 studies, showed no significant difference in FSE between single‐coil and dual‐coil ICD leads (OR: 1.03; 95% CI: [0.65–1.65]; *p* = 0.89; Figure S7).

### 3.6 Implant Time

**Figure 4:**
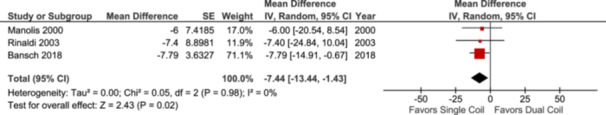
Forest plot of implant time.

Three studies reported on implant time, including data on 692 patients, the results of which showed single‐coil leads to be associated with a significantly lower implant time compared to dual‐coil leads (MD: −7.44; 95% CI [–13.44 to −1,43]; *p* = 0.02; Figure 4).

### 3.7 Cardiovascular Mortality

Four studies reported cardiac mortality, including data on 4625 patients. There was no significant difference in the cardiac mortality rate with single‐coil ICD leads in comparison to dual‐coil leads (OR: 0.90; 95% CI: [0.57–1.41]; *p* = 0.64; Figure S8).

A subgroup analysis was performed based on the study design. The analysis of three RCTs showed no significant difference in cardiac mortality rate with single‐coil ICD leads compared to dual‐coil leads (OR: 0.62; 95% CI: [0.31–1.24]; *p* = 0.18; Figure S9). The results were similar in the only prospective observational study to look at cardiac mortality (OR 1.40; 95% CI: [0.57–3.40]; *p* = 0.46; Figure S9). The single N‐RCT study also had no significant difference in cardiac mortality in single‐coil ICD leads when compared to dual‐coil leads (OR: 1.03; 95% CI: [0.46–2.30]; *p* = 0.94; Figure S9).

A further subgroup analysis was performed based on the number of patients. The analysis of the three studies involving more than 100 patients showed an insignificant difference in the cardiac mortality rate with single‐coil ICD leads in comparison to dual‐coil leads (OR 0.90; 95% CI: [0.55–1.48]; *p* = 0.68; Figure S10). This was similar to a single study involving < 100 patients (OR: 1.00; 95% CI: [0.06–16.59]; *p* = 1.00; Figure S10).

### 3.8 Lead Extraction

#### 3.8.1 Mortality

**Figure 5:**
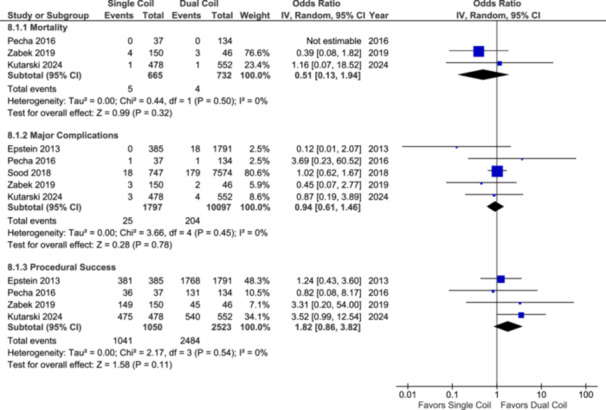
Forest plot of lead extraction‐related outcomes, including extraction‐related mortality, risk of major complications, and procedural success.

Three studies have reported mortality during lead extraction, including data on 1397 patients. The results showed no significant difference in mortality among single‐ versus dual‐coil leads (OR: 0.51; 95% CI: [0.13–1.94]; *p* = 0.32; Figure 5).

#### 3.8.2 Major Complications

Five studies reported major complications, including data on 11 894 patients. The results showed no significant difference in major complications with single‐coil leads compared to dual‐coil leads (OR: 0.94; 95% CI: [0.61–1.46]; *p* = 0.78; Figure 5).

#### 3.8.3 Procedural Success

Four studies reported on procedural success, including data on 3573 patients. The results showed no significant difference in the procedural success with single‐coil ICD leads in comparison to dual‐coil leads (OR: 1.82; 95% CI: [0.86–3.82]; *p* = 0.11; Figure 5).

## 4 Discussion

For individuals at heightened risk of potentially fatal cardiac events, ICDs are often recommended, especially in refractory cases. These devices play a crucial role in reducing the incidence of sudden cardiac death and unnecessary shocks by continuously monitoring heart rhythms and correcting electrical impulses [13, 14]. This meta‐analysis included 28 studies [15, 16, 17, 18, 19, 20, 21, 22, 23, 24, 25, 26, 27, 28, 29, 30, 31, 32, 33, 34, 35, 36, 37, 38, 39, 40, 41, 42], where we analyzed the data for DFT, all‐cause mortality, FSE, implant time, cardiovascular mortality, and lead extraction. Overall, the results of this quantitative synthesis revealed that dual‐coil ICDs had a significantly lower DFT, single‐coil ICDs had a significantly lower mortality rate, and the implant time was significantly shorter in single‐coil ICDs. Other outcomes, including FSE, cardiac mortality, postextraction major complications, postextraction procedural success, and post‐extraction mortality, did not demonstrate any significant statistical differences. However, it is noteworthy that in the RCTs, dual‐coil ICDs were still favored above the single‐coil arm, despite these conclusions.

The preference for dual‐coil ICDs persists despite the lack of significant evidence‐based superiority between the two types. This disparity underscores a critical aspect in clinical decision‐making. Although data suggest potential advantages of single‐coil ICDs, the preference for dual‐coil variants suggests a need for further elaboration. Factors influencing the choice of ICD type may include patient condition, device availability, or improved DFTs. Additionally, the consideration of dual‐coil ICD leads with superior vena cava (SVC) coil by Aoukar et al. did not yield notable benefits over conventional leads. Instead, it resulted in an increased lead complexity, expense, risk of failure, and difficulty in removal without enhancing outcomes for left‐sided implants [38]. A retrospective research study examined the correlation between the risk of significant problems and the difficulty of total lead extraction (TLE) and the presence of an SVC coil. Despite longer lead implant durations among the single‐coil leads, it was demonstrated that TLE of ICD leads with an SVC coil is linked to a 1.0% significant complication rate. In contrast, the removal of dual‐coil ICD leads was 2.6 times more challenging. Various adverse events have been observed with dual‐coil ICDs, including mortality, pericardial tamponade, respiratory failure, and RA/SVC tears [43].

According to research by Pokorney et al. [44], the utilization of dual‐coil ICDs declined significantly from 86% to 55% over a span of 5 years. The study also found that 72% of patients receiving dual‐coil ICDs were characterized by a higher likelihood of experiencing arrhythmias, and end‐stage kidney disease. Furthermore, the ALTITUDE study revealed a notable increase in the implantation rate of single‐coil ICDs over time, which was associated with a higher incidence of DFT [13, 40]. These findings are corroborated by research conducted by Leshem et al. [39] and a meta‐analysis by Sunderland et al. [43], which indicate a greater implantation rate of single‐coil leads than that of their dual‐coil counterparts. This preference is attributed to the reduced risk of adverse outcomes associated with single‐coil ICDs. Dual‐coil ICDs are associated with increased rates of complications, mortality, and procedural complexity, particularly in cases requiring extraction [39, 43].

Removal of dual‐coil ICDs has been associated with higher hazards, associated with the development of fibrosis at the sites where the lead comes into contact with the endocardium and vasculature. This may result in extensive vascular and myocardial adhesions and fibrous tissue ingrowth, both of which raise the risk of in‐hospital complications and mortality. Additionally, these risks may be influenced by factors such as lead design, diameter, and the location of the SVC coil. Due to their superior defibrillation capabilities, dual‐coil ICDs are more frequently chosen for patients who have a higher risk of serious cardiovascular disorders [13, 45, 46, 47].

Dual‐coil ICDs are considered superior to single‐coil systems in several key aspects. One primary advantage is the improved coverage of cardiac tissue. The additional coil, typically placed in the SVC, extends the defibrillation vector, ensuring a more comprehensive delivery of the electrical field during shocks. This broader coverage is particularly beneficial for patients with large hearts or those with complex cardiac anatomies. Furthermore, dual‐coil ICDs exhibit reduced shock impedance, facilitating a more efficient energy transfer during defibrillation, which can lead to lower DFTs and potentially reduce the energy required for successful shocks [44]. The flexibility in lead placement provided by dual‐coil systems allows for more tailored configurations, optimizing shock efficacy according to individual patient needs and anatomical considerations. Furthermore, having two coils offers a redundancy advantage if one coil fails, the other can continue to function, providing a critical backup that enhances the device's reliability and safety.

Our results are in‐line with numerous other studies that have established a connection between dual‐coil ICDs and a variety of unfavorable consequences. These include noticeably higher death rates, a rise in in‐hospital complications, longer implant durations, and increased complexity and risk involved in the extraction procedure [36, 37, 42]. Because of these difficulties, single‐coil ICDs are very advantageous in clinical settings due to their fewer risks and complications.

The DFT measures the minimum energy required for an ICD to effectively address life‐threatening arrhythmias. Achieving an optimal DFT is essential to prevent both ineffective shocks and sudden cardiac deaths [42]. Improvements in ICD technology, lead designs, and placement strategies have historically focused on optimizing DFTs [37]. Transitioning from epicardial to transvenous leads has proven to be beneficial by placing the leads closer to the heart's surface, resulting in lower DFTs. Dual‐coil leads, featuring defibrillation coils at both ends, offer enhanced shock coverage and lower DFTs, improving the probability of successfully terminating arrhythmias [42].

### 4.1 Clinical Implications

The decision on whether clinicians should prefer single‐coil versus dual‐coil ICDs involves a balance of efficacy, safety, and patient outcomes [43]. Recent studies have highlighted several considerations regarding this choice. Single‐coil ICDs are often favored for their reduced complication rates and lower incidence of lead‐related issues compared to dual‐coil ICDs. Research indicates that dual‐coil ICDs, while offering additional sensing capabilities and potential benefits in certain arrhythmic conditions, are associated with higher rates of lead fractures, infections, and other complications.

While our findings contradict earlier studies on the reduction of DFTs with dual‐coil ICDs compared to those by Sunderland et al. [43], they align with Kumar et al. [48]. This discrepancy may stem from differences in sample sizes across studies. Our analysis also indicates lower all‐cause mortality rates with single‐coil ICDs and no significant difference in FSE, consistent with prior meta‐analyses. There are various factors that potentially explain the increased mortality observed within the dual‐coil cohorts. First, the innately simpler design and lesser invasive placement of single‐coil ICDs permit patients to have a better postprocedural recovery, possibly leading to improved short‐term outcomes [41, 43]. Additionally, single‐coil ICDs are associated with better durability and longevity due to the lower risk of mechanical stress and wear linked to dual‐coil devices. This leads to a markedly lower lead failure rate among the single‐coil ICDs, ultimately reducing the necessity of follow‐up invasive medical procedures and thus improving mortality‐related outcomes in the respective cohort [49]. Furthermore, patients receiving dual‐coil ICDs may be more medically complex, potentially contributing to poorer outcomes compared to those with single‐coil devices due to the background health status of the patients. On the contrary, single‐coil ICDs remained superior in spite of patient adjustment in several studies, including factors such as age, sex, and implantation time, indicating that patient demographic factors may not be directly involved in this finding [29, 40]. Other features, including patient selection bias, evolving operator preferences, and unknown confounders, may also be involved. In conclusion, the all‐cause mortality findings are multifactorial in nature, with several integral aspects of both devices, as well as physician‐oriented elements, playing a significant role.

### 4.2 Limitations

Our meta‐analysis comparing single‐coil and dual‐coil ICD systems has several limitations. These include the pooling of diverse study designs, patient demographics, and comorbidities, which complicate the formation of consistent conclusions. Variations in ICD models and technologies, follow‐up durations, and potential publication biases may further impact the reliability of our findings. Methodological weaknesses in some studies, such as small sample sizes and inadequate control for confounding variables, also affect the strength of our conclusions. These limitations highlight the need for cautious clinical interpretation and emphasize the importance of further research, especially into homogeneous patient populations, to validate our findings and address such challenges.

## 5 Conclusion

The choice between single‐ and dual‐coil ICDs requires a comprehensive evaluation of efficacy, safety, and patient well‐being. As per the results of our quantitative synthesis, dual‐coil ICDs demonstrated greater efficacy through DFTs, whereas single‐coil ICDs illustrated significantly lower mortality rates and lead implant times. Collectively, dual‐coil ICDs possess greater efficacy potential, whereas single‐coil ICDs boast a comparatively superior safety profile. In terms of lead extraction‐related outcomes, our analysis could not detect any statistically significant differences between the two systems. Dual‐coil ICDs continue to remain as the preferred choice among physicians and cardiologists, largely due to their perceived advantages in complex clinical situations. In conclusion, the selection of an ICD device must involve a multifaceted evaluation of patient case, the complexity of their pathology, and the necessity of backup pacing, especially in recurrent arrhythmic conditions. However, for simpler cases, single‐coil ICDs continue to hold merit due to their safer profile. As ICD technology continues to evolve, rigorous clinical trials and detailed performance assessments will be crucial in guiding device selection, minimizing complications, and ultimately improving patient outcomes.

## Ethics Statement

The authors have nothing to report.

## Conflicts of Interest

The authors declare no conflicts of interest.

## Supporting information

Supporting information.

## Data Availability

The data that supports the findings of this study are available in the Supporting Information of this article.
