# Supplementary material for: Comparison of Single‐Coil Versus Dual‐Coil Implantable Cardioverter Defibrillator Devices: A Systematic Review and Meta‐Analysis of Efficacy and Extraction‐Related Outcomes
Source: Clin Cardiol. 2025 Feb 5;48(2):e70083. doi: 10.1002/clc.70083 (PMC11799043; doi:10.1002/clc.70083)
Supplement: Supplementary file 1 — Supporting information. [file CLC-48-e70083-s001.pdf]

Database	Search Strategy	Number of Studies
**PubMed**	((("single person"[MeSH Terms] OR ("single"[All Fields] AND "person"[All Fields]) OR "single person"[All Fields] OR "single"[All Fields] OR "singles"[All Fields]) AND "coil"[All Fields]) OR ("dual"[All Fields] AND "coil"[All Fields]) OR (("double"[All Fields] OR "doubled"[All Fields] OR "doubles"[All Fields] OR "doubling"[All Fields] OR "doublings"[All Fields]) AND "coil"[All Fields]) OR (("single person"[MeSH Terms] OR ("single"[All Fields] AND "person"[All Fields]) OR "single person"[All Fields] OR "single"[All Fields] OR "singles"[All Fields]) AND ("chamber"[All Fields] OR "chamber s"[All Fields] OR "chambered"[All Fields] OR "chambers"[All Fields])) OR ("dual"[All Fields] AND ("chamber"[All Fields] OR "chamber s"[All Fields] OR "chambered"[All Fields] OR "chambers"[All Fields]))) AND ("defibrillators, implantable"[MeSH Terms] OR ("defibrillators"[All Fields] AND "implantable"[All Fields]) OR "implantable defibrillators"[All Fields] OR ("implantable"[All Fields] AND "defibrillator"[All Fields]) OR "implantable defibrillator"[All Fields] OR ("defibrillators, implantable"[MeSH Terms] OR ("defibrillators"[All Fields] AND "implantable"[All Fields]) OR "implantable defibrillators"[All Fields] OR ("implantable"[All Fields] AND "cardioverter"[All Fields] AND "defibrillator"[All Fields]) OR "implantable cardioverter defibrillator"[All Fields]) OR "ICD"[All Fields]) AND ((("atrialisation"[All Fields] OR "atrialization"[All Fields] OR "atrialized"[All Fields] OR "atrially"[All Fields] OR "heart atria"[MeSH Terms] OR ("heart"[All Fields] AND "atria"[All Fields]) OR "heart atria"[All Fields] OR "atrial"[All Fields]) AND ("tachyarrhythmia s"[All Fields] OR "tachycardia"[MeSH Terms] OR "tachycardia"[All Fields] OR "tachyarrhythmia"[All Fields] OR "tachyarrhythmias"[All Fields])) OR ("arrhythmia s"[All Fields] OR "arrhythmias, cardiac"[MeSH Terms] OR ("arrhythmias"[All Fields] AND "cardiac"[All Fields]) OR "cardiac arrhythmias"[All Fields] OR "arrhythmia"[All Fields] OR "arrhythmias"[All Fields]) OR ("ventricular fibrillation"[MeSH Terms] OR ("ventricular"[All Fields] AND "fibrillation"[All Fields]) OR "ventricular fibrillation"[All Fields]) OR ("heart arrest"[MeSH Terms] OR ("heart"[All Fields] AND "arrest"[All Fields]) OR "heart arrest"[All Fields] OR ("cardiac"[All Fields] AND "arrest"[All Fields]) OR "cardiac arrest"[All Fields]) OR ("cardiomyopathy, hypertrophic"[MeSH Terms] OR ("cardiomyopathy"[All Fields] AND "hypertrophic"[All Fields]) OR "hypertrophic cardiomyopathy"[All Fields] OR ("hypertrophic"[All Fields] AND "cardiomyopathy"[All Fields])) OR ("health cost manage"[Journal] OR "health care manag frederick"[Journal] OR "hcm"[All Fields]))	1,002
**Cochrane Library**	(single coil OR dual coil OR double coil OR single chamber OR dual chamber) AND (implantable defibrillator OR implantable cardioverter defibrillator OR ICD) AND (atrial tachyarrhythmias OR arrhythmia OR ventricular fibrillation OR cardiac arrest OR hypertrophic cardiomyopathy OR HCM)	195
**ScienceDirect**	(“single coil” OR “dual coil”) AND (“implantable cardioverter defibrillators” OR “ICD”) AND (“arrhythmia” OR “hypertrophic cardiomyopathy” OR “ventricular fibrillation” OR “cardiac arrest”)	574

Supplementary Table 1. Detailed search strategy used for systematic review.

Study Title	Total Number of Patients, n	Follow-up Time, months	Data Source	Study Design	Device Type	Number of Patients in Each Cohort, n		Coronary Artery Disease, n (%)	
						Single-Coil	Dual-Coil	Single-Coil	Dual-Coil
**Kutarski 2024**	1,030	77.04	Single high-volume center	Retrospective Cohort	ICD	478	552	272 (57%)	324 (59%)
**Larsen 2019**	216	NR	Single-Center (The Department of Cardiology at Aalborg University Hospital in Denmark)	RCT	ICD (Multiple Manufacturers)	106	110	74 (70%)	59 (54%)
**Zabek 2019**	196	1.0	CRT-D	Prospective Cohort	SC and DC ICD	150	46	100 (67%)	29 (63%)
**Bansch 2018**	1,067	23.9	NORDIC ICD	RCT	SC and DC ICD	517	550	320 (62%)	374 (68%)
**Sood 2018**	8,321	NR	NCDR ICD Registry	Retrospective Cohort	ICD	747	7,574	NR	NR
**Leshem 2017**	2,673	20.2	Israeli ICD Registry.	Prospective Cohort	SC/DC-ICD, CRT-D	357	2,316	920 (64)	1639 (65)
**Pecha 2016**	171	1.0	Retrospective Database	Retrospective Cohort	SC/DC ICD	37	134	NR	NR
**Larsen 2016**	4,769	72.0	3 Danish national registries	Retrospective Cohort	SC/DC-ICD, CRT-D (Multiple manufacturers)	1,854	2,915	67 (3.6%)	69 (2.4%)
**Baccillieri 2015**	469	NR	Italian universities consortium ICD registry	Prospective Cohort	SC/DC-ICD, CRT-D (Multiple manufacturers)	311	158	NR	NR
**Hsu 2015**	129,520	60.0	ALTITUDE	Prospective Cohort	SC/DC-ICD, CRT-D Boston	19,190	110,330	NR	NR
**Kutyifa 2013**	1,783	39.6	MADIT-CRT	N-RCT	SC/DC-ICD, CRT-D Boston	162	1,621	51 (31%)	709 (45%)
**Aoukar 2013**	809	45.5	SCD-HeFT	N-RCT	SC-ICD, Medtronic	246	563	NR	NR
**Epstein 2013**	2,176	NR	NR	Retrospective Cohort	SC/DC-ICD	385	1,791	NR	NR
**Ellis 2012**	77	24.0	St. Jude ACT ICD registry	Retrospective Cohort	SC/DC-ICD, CRT-D, St. Jude	22	55	NR	NR
**Gold 2008**	113	NR	Multiple US centers	RCT	SC-ICD, St. Jude	113	113	86 (76%)	
**Varma 2008**	42	NR	Single US center	Prospective Cohort	SC/DC-ICD, St. Jude	42	42	28 (67%)	
**Keane 2007**	16	NR	Informal Prospective Registry	Retrospective Cohort	SC/DC ICD	16	16	NR	NR
**Andrzej 2005**	138	NR	Patient Records (Department of Cardiology-Medical Academy in Gdañsk.)	Prospective Cohort	ICD	76	62	46 (61%)	39 (63%)
**Rubb 2004**	11	NR	Single German center	RCT	DC-ICD, Medtronic	11	11	10 (91%)	
**Rinaldi 2003**	76	NR	Multiple European centers	RCT	SC-ICD, Guidant	38	38	28 (74%)	28 (74%)

**Schulte 2001**	80	NR	Single German center	RCT	SC-ICD, Guidant or Medtronic	40	40	48%	55%
**Andraghetti 2001**	500	NR	NR	N-RCT	NR	132	368	NR	NR
**Libero 2001**	35	NR	Multiple European centers	RCT	SC/DC-ICD, Guidant	35	35	22 (63%)	
**Gold 2000**	27	NR	Multiple US centers	RCT	DC-ICD, Medtronic	27	27	19 (70%)	
**Manolis 2000**	94	NR	Single Greek center	N-RCT	SC-ICD, (Multiple manufacturers)	32	62	60 (64%)	
**Gold 1998**	50	NR	Single US center	Prospective Cohort	ICD, Cardiac Pacemakers Inc.	50	50	NR	NR
**Gold 1997**	21	NR	Single US center	Prospective Cohort	ICD, Cardiac Pacemakers Inc.	21	21	16 (76%)	
**Bardy 1994**	15	NR	Single US center	RCT	ICD, Medtronic	15	15	9 (60%)	

n = Number of Patients; RCT = Randomized Controlled Trial; N-RCT = Non-Randomized Controlled Trial, ICD = Implantable Cardioverter Defibrillator device.

Supplementary Table 2. Baseline clinical and study characteristics of the included study population.

	S1	S2	S3	S4	C1	C2	O1	O2	O3	Total
**Baccillieri 2010**	*	*	*	*	*	*	*	*	*	**9/9**
**Gold 1997**	*	*	*	*	*		*	*	*	**8/9**
**Gold 1998**	*	*	*	*	*		*	*	*	**8/9**
**Hsu 2014**	*	*	*	*	*	*	*	*	*	**9/9**
**Larsen 2016**	*	*	*	*	*	*	*	*	*	**9/9**
**Leshem 2017**	*	*	*	*	*	*	*	*	*	**9/9**
**Zabek 2019**	*	*	*	*	*	*	*	*	*	**9/9**
**Andrzej 2005**	*	*	*	*	*	*	*	*	*	**9/9**
**Ellis 2012**	*	*	*	*	*		*	*	*	**8/9**
**Varma 2008**	*	*	*	*	*		*	*	*	**8/9**
**Keane 2007**	*	*	*	*	*	*	*	*	*	**9/9**
**Pecha 2016**	*	*	*	*			*	*	*	**7/9**
**Sood 2007**	*	*	*	*	*	*	*	*	*	**9/9**
**Kutarski 2024**	*	*	*	*	*	*	*	*	*	**9/9**
**Epstein 2013**	*	*	*	*	*	*	*	*	*	**9/9**

Supplementary Table 3. Author judgements for risk of bias assessments using the Cochrane Risk of Bias Tool for Cohort Studies.

	D1	D2	D3	D4	D5	D6	D7	Total
**Andraghetti 2001**	Serious	Low	Low	Moderate	Low	Moderate	Low	Low
**Aoukar 2013**	Critical	Low	Critical	Low	Low	Serious	Low	Moderate
**Kutfiya 2013**	Serious	Critical	Serious	Low	Low	Critical	Low	Serious
**Manolis 2000**	Low	Critical	Low	Serious	Low	Moderate	Moderate	Moderate

Supplementary Table 4. Author judgements for risk of bias assessments using the ROBINS-I tool for non-randomized studies.

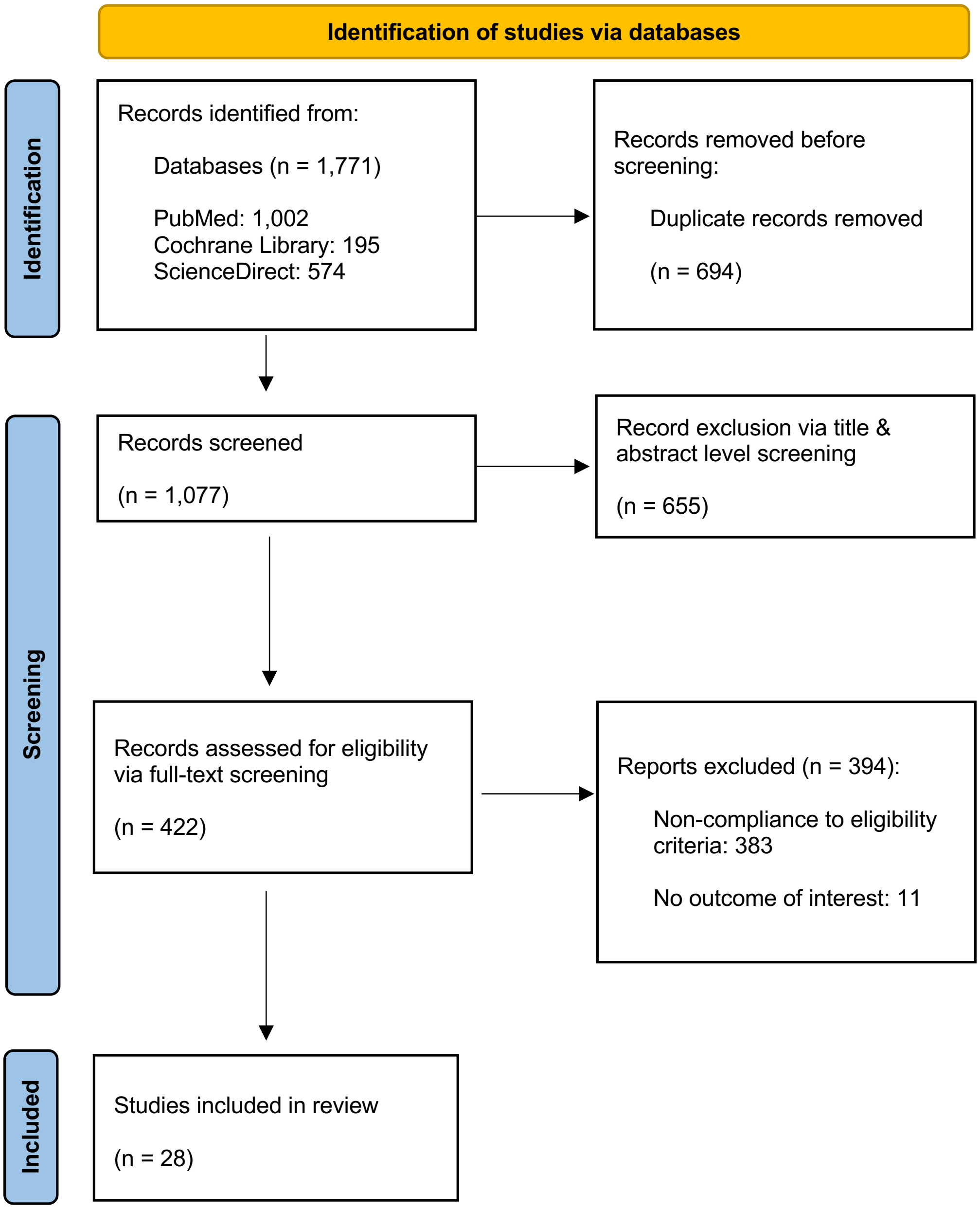

Supplementary Figure 1. PRISMA flowchart depicting the screening process.

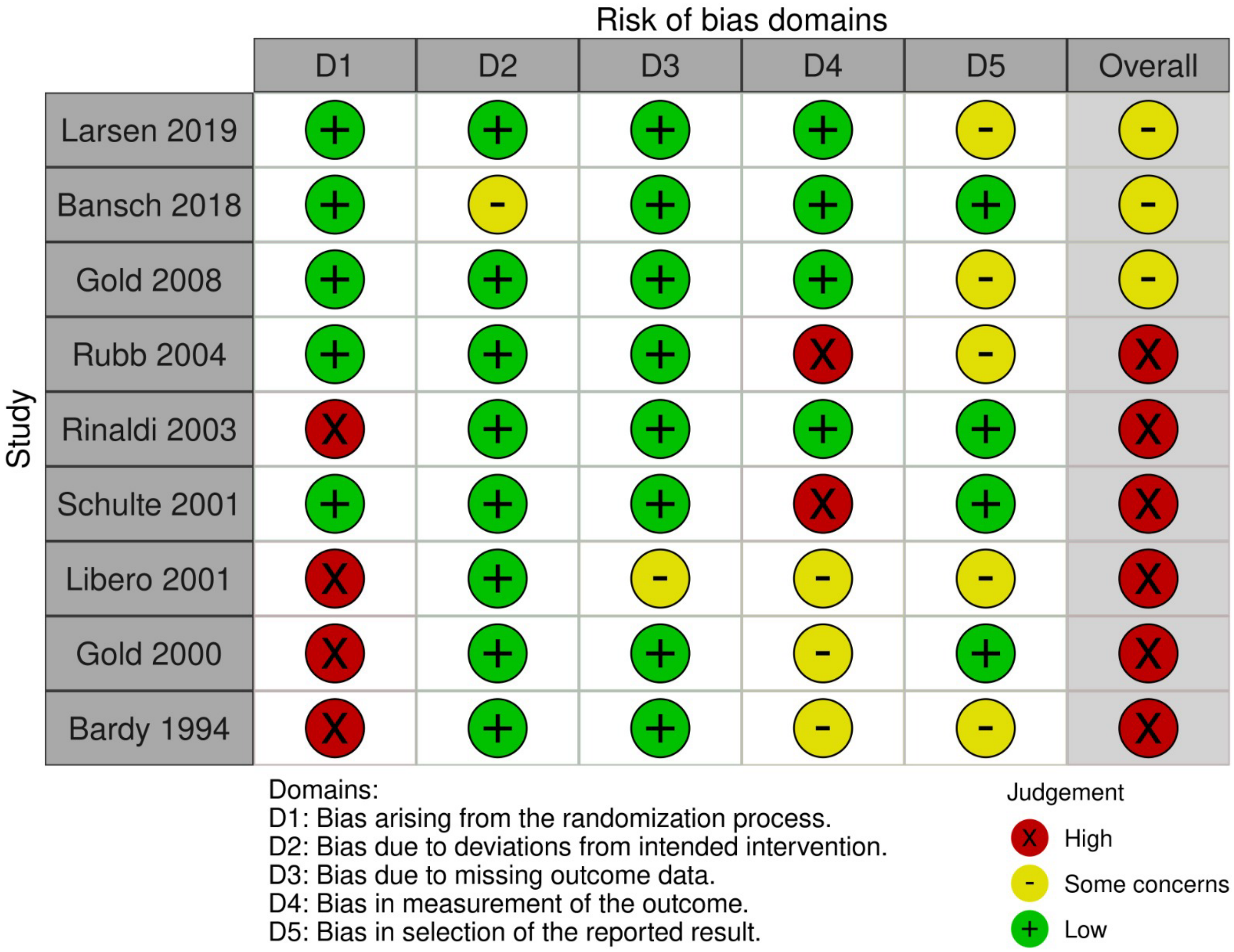

Supplementary Figure 2. Author judgements for risk of bias assessments using the Cochrane Risk of Bias Tool (RoB 2.0) for Randomized Controlled Trials.

	Single Coil			Dual Coil				Mean Difference	
**Study or Subgroup**	**Mean**	**SD**	**Total**	**Mean**	**SD**	**Total**	**Weight**	**IV, Random, 95% CI**	**Year**
**1.1.3 RCT**									
Bardy 1994	7.4	5.2	15	6	3.4	15	2.1%	1.40 [-1.74, 4.54]	1994
Gold 2000	11.2	6.6	27	8.5	4.2	27	2.4%	2.70 [-0.25, 5.65]	2000
Schulte 2001	8.4	3.7	40	8	3.6	40	5.5%	0.40 [-1.20, 2.00]	2001
Libero 2001	10.4	4.3	44	8.6	4	44	5.0%	1.80 [0.06, 3.54]	2001
Rinaldi 2003	10.25	4.07	38	10.22	5.17	38	4.0%	0.03 [-2.06, 2.12]	2003
Rubb 2004	12	5.6	11	16.3	7.8	11	0.8%	-4.30 [-9.97, 1.37]	2004
Gold 2008	10.4	5.7	113	9.8	5.6	113	6.0%	0.60 [-0.87, 2.07]	2008
Bansch 2018	17.46	5.43	516	16.09	3.25	551	10.6%	1.37 [0.83, 1.91]	2018
**Subtotal (95% CI)**			**804**			**839**	**36.2%**	**1.08 [0.48, 1.69]**	
Heterogeneity: Tau² = 0.12; Chi² = 8.25, df = 7 (P = 0.31); I² = 15%									
Test for overall effect: Z = 3.52 (P = 0.0004)									
**1.1.4 N-RCT**									
Manolis 2000	9.3	3.6	32	10.2	3.7	62	5.7%	-0.90 [-2.45, 0.65]	2000
Andraghetti 2001	6.3	3.1	368	6.9	3.7	132	9.7%	-0.60 [-1.31, 0.11]	2001
Kutyifa 2013	19.4	6.1	162	17.6	5.8	1621	8.3%	1.80 [0.82, 2.78]	2013
Aoukar 2013	13.4	23.69	246	12.5	21.42	563	1.8%	0.90 [-2.55, 4.35]	2013
**Subtotal (95% CI)**			**808**			**2378**	**25.5%**	**0.23 [-1.30, 1.77]**	
Heterogeneity: Tau² = 1.79; Chi² = 17.12, df = 3 (P = 0.0007); I² = 82%									
Test for overall effect: Z = 0.30 (P = 0.77)									
**1.1.5 Prospective Observational**									
Gold 1997	10.1	5.2	21	11.2	3.4	21	2.8%	-1.10 [-3.76, 1.56]	1997
Gold 1998	10.1	5	50	8.7	4	50	4.9%	1.40 [-0.37, 3.17]	1998
Andrzej 2005	11.45	5.25	76	9.8	4.6	62	5.3%	1.65 [0.01, 3.29]	2005
Keane 2007	34	4	16	36.4	2.2	16	3.6%	-2.40 [-4.64, -0.16]	2007
Varma 2008	19.5	9.2	42	18.7	8.6	42	1.5%	0.80 [-3.01, 4.61]	2008
**Subtotal (95% CI)**			**205**			**191**	**18.1%**	**0.15 [-1.50, 1.81]**	
Heterogeneity: Tau² = 2.13; Chi² = 10.72, df = 4 (P = 0.03); I² = 63%									
Test for overall effect: Z = 0.18 (P = 0.86)									
**1.1.6 Retrospective Observational**									
Baccillieri 2010	18.9	4.8	311	19	5.1	158	8.4%	-0.10 [-1.06, 0.86]	2010
Larsen 2016	23.2	4.3	1854	22.1	3.9	2915	11.7%	1.10 [0.86, 1.34]	2016
**Subtotal (95% CI)**			**2165**			**3073**	**20.1%**	**0.59 [-0.57, 1.75]**	
Heterogeneity: Tau² = 0.59; Chi² = 5.67, df = 1 (P = 0.02); I² = 82%									
Test for overall effect: Z = 1.00 (P = 0.32)									
**Total (95% CI)**			**3982**			**6481**	**100.0%**	**0.58 [0.07, 1.09]**	
Heterogeneity: Tau² = 0.56; Chi² = 54.06, df = 18 (P < 0.0001); I² = 67%									
Test for overall effect: Z = 2.25 (P = 0.02)									
Test for subgroup differences: Chi² = 2.05, df = 3 (P = 0.56), I² = 0%									

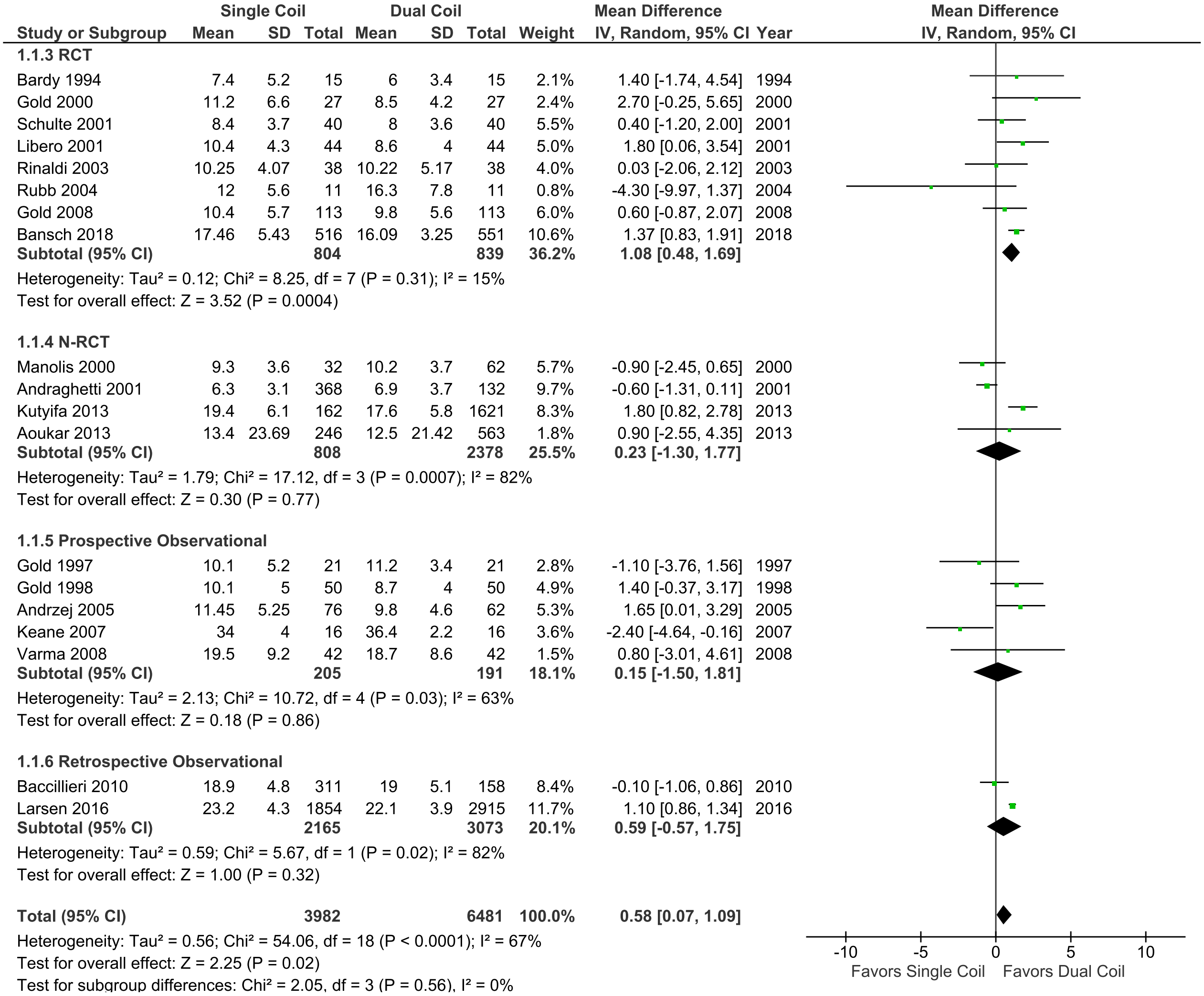

Supplementary Figure 3. Subgroup analysis on basis of study design for defibrillation threshold (DFT).

Study or Subgroup	Single Coil Mean	Single Coil SD	Single Coil Total	Dual Coil Mean	Dual Coil SD	Dual Coil Total	Weight	Mean Difference IV, Random, 95% CI	Year
**1.1.1 More than 100**									
Andraghetti 2001	6.3	3.1	368	6.9	3.7	132	9.8%	-0.60 [-1.31, 0.11]	2001
Andrzej 2005	11.45	5.25	76	9.8	4.6	62	5.4%	1.65 [0.01, 3.29]	2005
Gold 2008	10.4	5.7	113	9.8	5.6	113	6.0%	0.60 [-0.87, 2.07]	2008
Baccillieri 2010	18.9	4.8	311	19	5.1	158	8.5%	-0.10 [-1.06, 0.86]	2010
Aoukar 2013	13.4	23.69	246	12.5	21.42	563	1.9%	0.90 [-2.55, 4.35]	2013
Kutyifa 2013	19.4	6.1	162	17.6	5.8	1621	8.3%	1.80 [0.82, 2.78]	2013
Larsen 2016	23.2	4.3	1854	22.1	3.9	2915	11.8%	1.10 [0.86, 1.34]	2016
Bansch 2018	17.46	5.43	516	16.09	3.25	551	10.6%	1.37 [0.83, 1.91]	2018
**Subtotal (95% CI)**			**3646**			**6115**	**62.2%**	**0.80 [0.20, 1.40]**	
Heterogeneity: Tau² = 0.46; Chi² = 30.64, df = 7 (P < 0.0001); I² = 77%									
Test for overall effect: Z = 2.61 (P = 0.009)									
**1.1.2 Less than 100**									
Bardy 1994	7.4	5.2	15	6	3.4	15	2.2%	1.40 [-1.74, 4.54]	1994
Gold 1997	10.1	5.2	21	11.2	3.4	21	2.8%	-1.10 [-3.76, 1.56]	1997
Gold 1998	10.1	5	50	8.7	4	50	4.9%	1.40 [-0.37, 3.17]	1998
Gold 2000	11.2	6.6	27	8.5	4.2	27	2.4%	2.70 [-0.25, 5.65]	2000
Manolis 2000	9.3	3.6	32	10.2	3.7	62	5.7%	-0.90 [-2.45, 0.65]	2000
Libero 2001	10.4	4.3	35	8.6	4	35	4.4%	1.80 [-0.15, 3.75]	2001
Schulte 2001	8.4	3.7	40	8	3.6	40	5.5%	0.40 [-1.20, 2.00]	2001
Rinaldi 2003	10.25	4.07	38	10.22	5.17	38	4.0%	0.03 [-2.06, 2.12]	2003
Rubb 2004	12	5.6	11	16.3	7.8	11	0.8%	-4.30 [-9.97, 1.37]	2004
Keane 2007	34	4	16	36.4	2.2	16	3.6%	-2.40 [-4.64, -0.16]	2007
Varma 2008	19.5	9.2	42	18.7	8.6	42	1.6%	0.80 [-3.01, 4.61]	2008
**Subtotal (95% CI)**			**327**			**357**	**37.8%**	**0.20 [-0.75, 1.15]**	
Heterogeneity: Tau² = 1.08; Chi² = 18.31, df = 10 (P = 0.05); I² = 45%									
Test for overall effect: Z = 0.41 (P = 0.68)									
**Total (95% CI)**			**3973**			**6472**	**100.0%**	**0.57 [0.06, 1.08]**	
Heterogeneity: Tau² = 0.56; Chi² = 53.85, df = 18 (P < 0.0001); I² = 67%									
Test for overall effect: Z = 2.21 (P = 0.03)									
Test for subgroup differences: Chi² = 1.11, df = 1 (P = 0.29), I² = 9.7%									

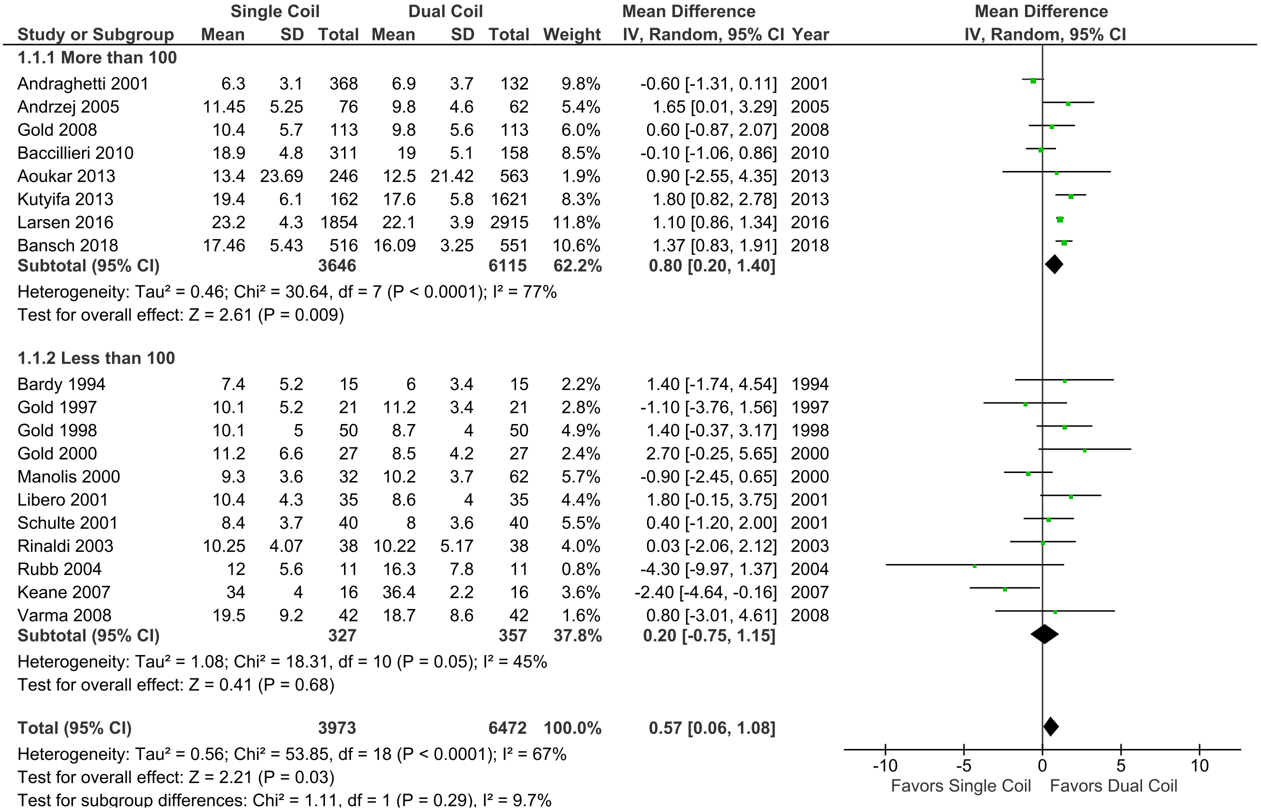

Supplementary Figure 4. Subgroup analysis on basis of number of patients for defibrillation threshold (DFT).

Study or Subgroup	log[Hazard Ratio]	SE	Weight	Hazard Ratio IV, Random, 95% CI
**1.2.3 RCT**				
Bansch 2018	0.2168	0.2506	1.2%	1.24 [0.76, 2.03]
**Subtotal (95% CI)**			**1.2%**	**1.24 [0.76, 2.03]**
Heterogeneity: Not applicable				
Test for overall effect: Z = 0.87 (P = 0.39)				
**1.2.4 N-RCT**				
Aoukar 2013	-0.0465	0.173	2.6%	0.95 [0.68, 1.34]
Kutyifa 2013	0.0914	0.3246	0.7%	1.10 [0.58, 2.07]
**Subtotal (95% CI)**			**3.3%**	**0.98 [0.73, 1.33]**
Heterogeneity: Tau² = 0.00; Chi² = 0.14, df = 1 (P = 0.71); I² = 0%				
Test for overall effect: Z = 0.10 (P = 0.92)				
**1.2.5 Prospective Observational**				
Hsu 2014	-0.0906	0.0307	81.5%	0.91 [0.86, 0.97]
Leshem 2017	0.2507	0.2482	1.2%	1.28 [0.79, 2.09]
**Subtotal (95% CI)**			**82.8%**	**0.99 [0.75, 1.32]**
Heterogeneity: Tau² = 0.03; Chi² = 1.86, df = 1 (P = 0.17); I² = 46%				
Test for overall effect: Z = 0.06 (P = 0.95)				
**1.2.6 Retrospective Observational**				
Larsen 2016	-0.1624	0.0777	12.7%	0.85 [0.73, 0.99]
**Subtotal (95% CI)**			**12.7%**	**0.85 [0.73, 0.99]**
Heterogeneity: Not applicable				
Test for overall effect: Z = 2.09 (P = 0.04)				
**Total (95% CI)**			**100.0%**	**0.91 [0.87, 0.97]**
Heterogeneity: Tau² = 0.00; Chi² = 4.63, df = 5 (P = 0.46); I² = 0%				
Test for overall effect: Z = 3.22 (P = 0.001)				
Test for subgroup differences: Chi² = 2.91, df = 3 (P = 0.41), I² = 0%				

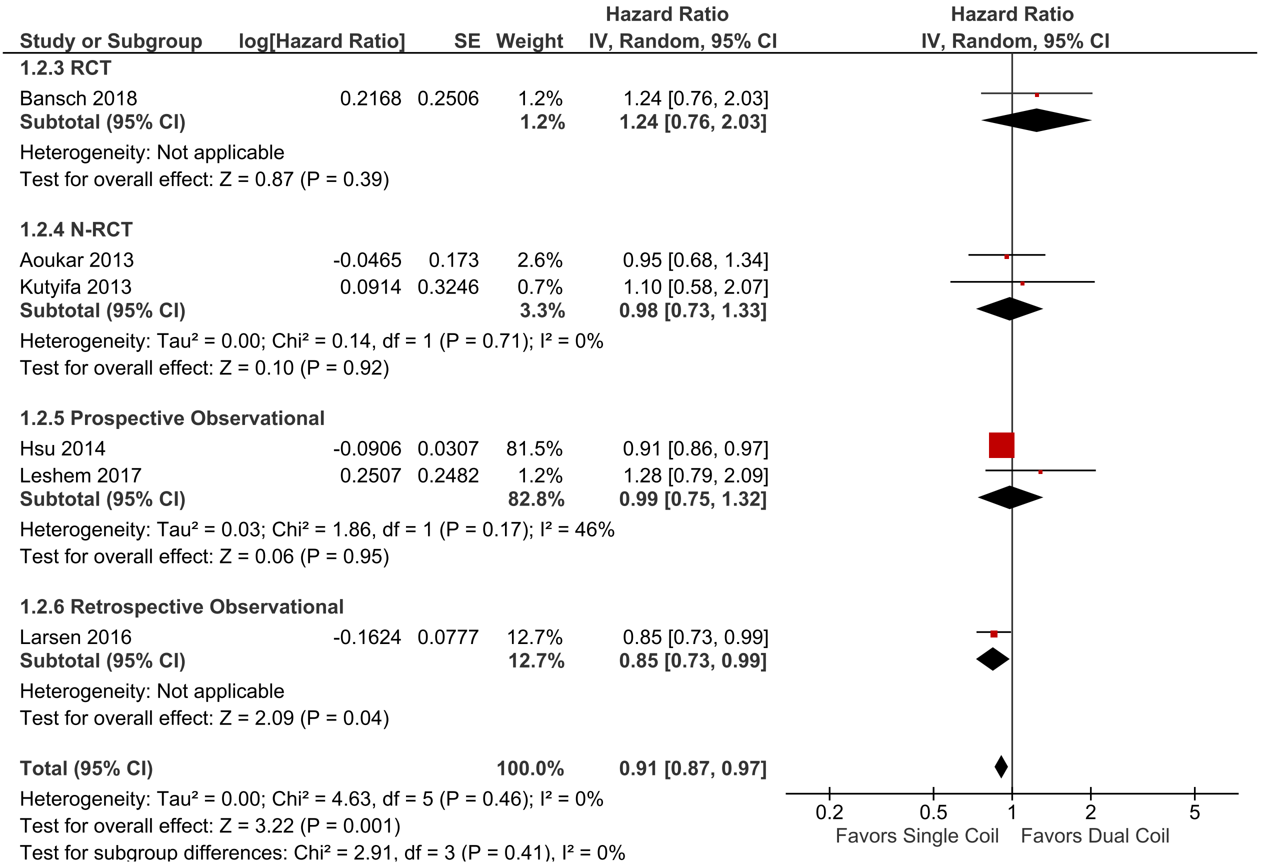

Supplementary Figure 5. Subgroup analysis on basis of study design for all-cause mortality.

Study or Subgroup	log[Odds Ratio]	SE	Weight	Odds Ratio IV, Random, 95% CI	Year
**1.2.1 RCT**					
Bansch 2018	0.6783	0.3185	24.6%	1.97 [1.06, 3.68]	2018
Larsen 2019	0.1974	0.3636	21.5%	1.22 [0.60, 2.48]	2019
**Subtotal (95% CI)**			**46.0%**	**1.60 [1.00, 2.56]**	
Heterogeneity: Tau² = 0.00; Chi² = 0.99, df = 1 (P = 0.32); I² = 0%					
Test for overall effect: Z = 1.96 (P = 0.05)					
**1.2.2 N-RCT**					
Aoukar 2013	-0.9034	0.525	13.5%	0.41 [0.14, 1.13]	2013
Kutiyafa 2013	-0.3345	1.0717	4.1%	0.72 [0.09, 5.85]	2013
**Subtotal (95% CI)**			**17.6%**	**0.45 [0.18, 1.14]**	
Heterogeneity: Tau² = 0.00; Chi² = 0.23, df = 1 (P = 0.63); I² = 0%					
Test for overall effect: Z = 1.68 (P = 0.09)					
**1.2.3 Prospective Observational**					
Hsu 2015	0.0807	0.4188	18.2%	1.08 [0.48, 2.46]	2015
**Subtotal (95% CI)**			**18.2%**	**1.08 [0.48, 2.46]**	
Heterogeneity: Not applicable					
Test for overall effect: Z = 0.19 (P = 0.85)					
**1.2.4 Retrospective Observational**					
Ellis 2012	-0.8916	1.0999	3.9%	0.41 [0.05, 3.54]	2012
Larsen 2016	-0.3567	0.5053	14.2%	0.70 [0.26, 1.88]	2016
**Subtotal (95% CI)**			**18.2%**	**0.64 [0.26, 1.57]**	
Heterogeneity: Tau² = 0.00; Chi² = 0.20, df = 1 (P = 0.66); I² = 0%					
Test for overall effect: Z = 0.98 (P = 0.33)					
**Total (95% CI)**			**100.0%**	**1.00 [0.64, 1.57]**	
Heterogeneity: Tau² = 0.11; Chi² = 8.81, df = 6 (P = 0.18); I² = 32%					
Test for overall effect: Z = 0.01 (P = 0.99)					
Test for subgroup differences: Chi² = 7.40, df = 3 (P = 0.06), I² = 59.5%					

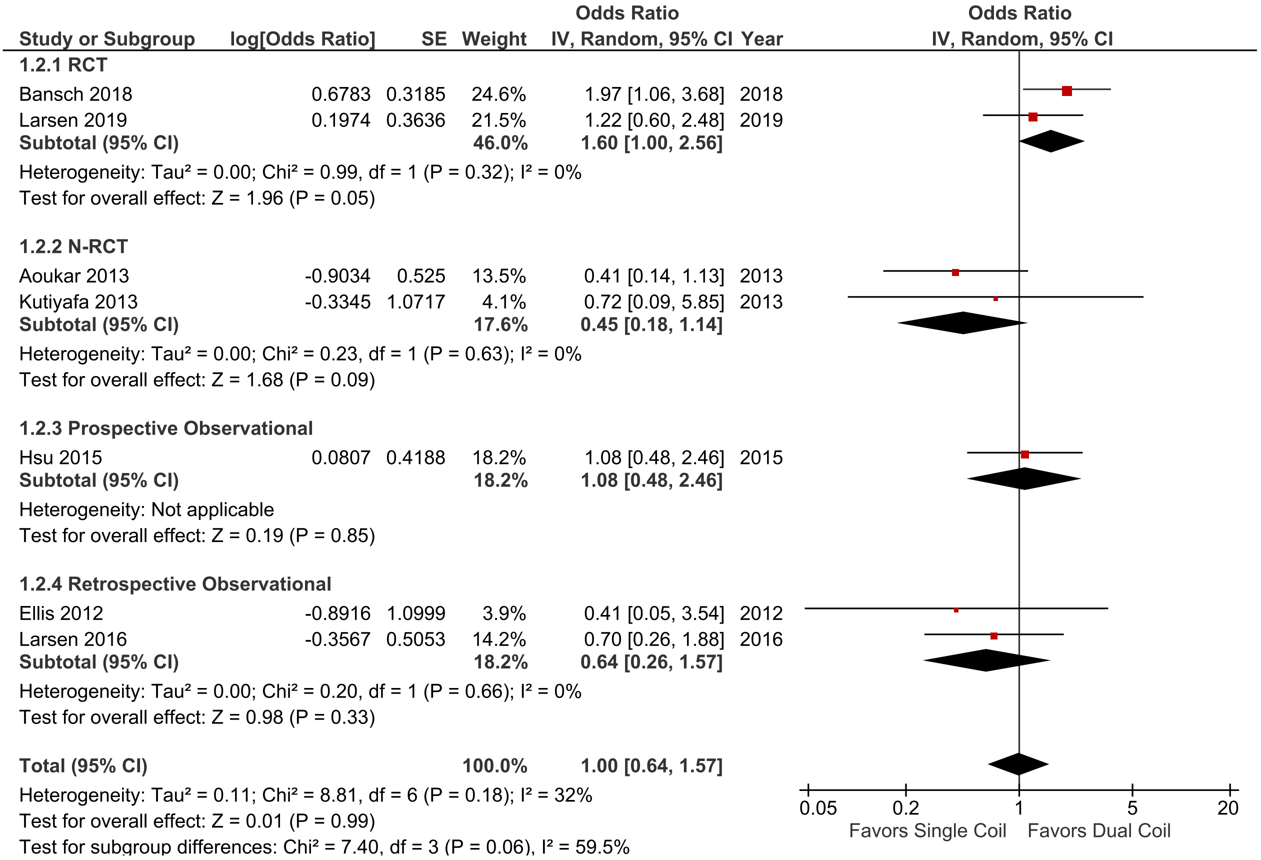

Supplementary Figure 6. Subgroup analysis on basis of study design for first-shock efficacy.

Study or Subgroup	log[Odds Ratio]	SE	Weight	Odds Ratio IV, Random, 95% CI	Year
**1.2.1 More than 100**					
Aoukar 2013	-0.9034	0.525	13.5%	0.41 [0.14, 1.13]	2013
Kutiyafa 2013	-0.3345	1.0717	4.1%	0.72 [0.09, 5.85]	2013
Hsu 2015	0.0807	0.4188	18.2%	1.08 [0.48, 2.46]	2015
Larsen 2016	-0.3567	0.5053	14.2%	0.70 [0.26, 1.88]	2016
Bansch 2018	0.6783	0.3185	24.6%	1.97 [1.06, 3.68]	2018
Larsen 2019	0.1974	0.3636	21.5%	1.22 [0.60, 2.48]	2019
**Subtotal (95% CI)**			**96.1%**	**1.03 [0.65, 1.65]**	
Heterogeneity: Tau² = 0.12; Chi² = 8.00, df = 5 (P = 0.16); I² = 37%					
Test for overall effect: Z = 0.14 (P = 0.89)					
**1.2.2 Less than 100**					
Ellis 2012	-0.8916	1.0999	3.9%	0.41 [0.05, 3.54]	2012
**Subtotal (95% CI)**			**3.9%**	**0.41 [0.05, 3.54]**	
Heterogeneity: Not applicable					
Test for overall effect: Z = 0.81 (P = 0.42)					
**Total (95% CI)**			**100.0%**	**1.00 [0.64, 1.57]**	
Heterogeneity: Tau² = 0.11; Chi² = 8.81, df = 6 (P = 0.18); I² = 32%					
Test for overall effect: Z = 0.01 (P = 0.99)					
Test for subgroup differences: Chi² = 0.68, df = 1 (P = 0.41), I² = 0%					

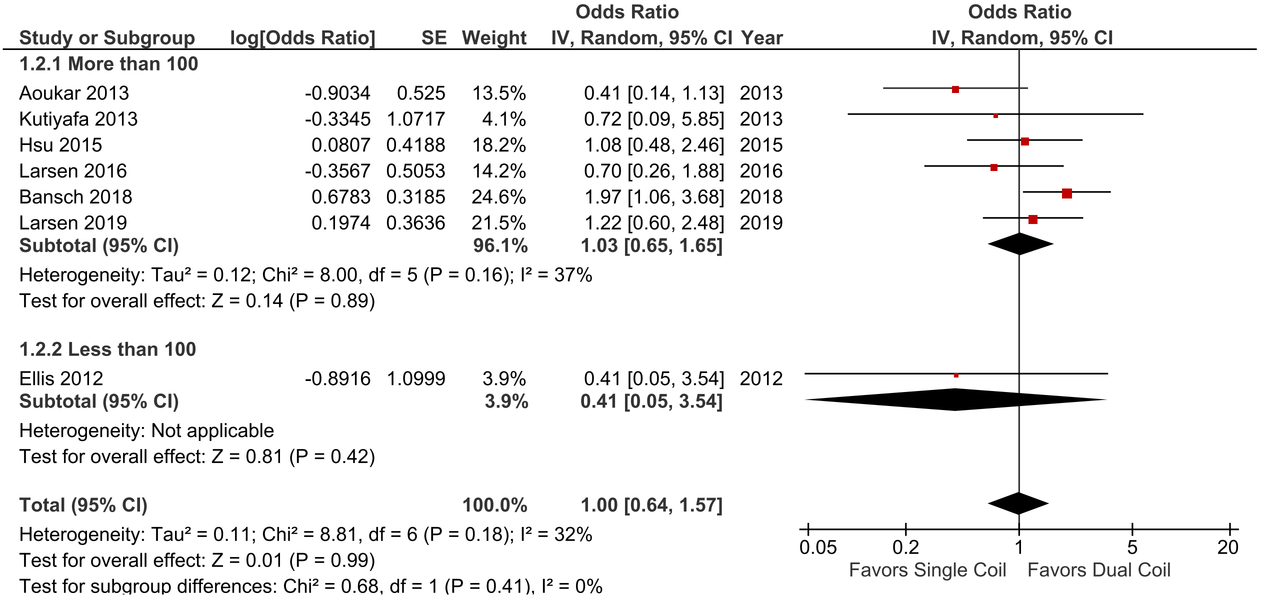

Supplementary Figure 7. Subgroup analysis on basis of number of patients for first-shock efficacy.

	Single Coil		Dual Coil			Odds Ratio	
**Study or Subgroup**	**Events**	**Total**	**Events**	**Total**	**Weight**	**IV, Random, 95% CI**	**Year**
Rinaldi 2003	1	38	1	38	2.6%	1.00 [0.06, 16.59]	2003
Aoukar 2013	9	246	20	563	31.9%	1.03 [0.46, 2.30]	2013
Leshem 2017	6	357	28	2316	25.9%	1.40 [0.57, 3.40]	2017
Bansch 2018	12	517	21	550	39.6%	0.60 [0.29, 1.23]	2018
**Total (95% CI)**		**1158**		**3467**	**100.0%**	**0.90 [0.57, 1.41]**	
Total events	28		70				

Heterogeneity: Tau² = 0.00; Chi² = 2.29, df = 3 (P = 0.51); I² = 0%

Test for overall effect: Z = 0.46 (P = 0.64)

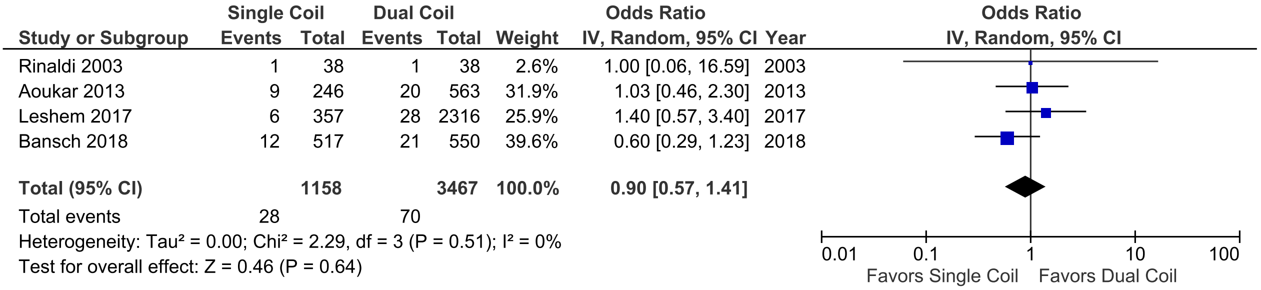

Supplementary Figure 8. Forest plot of cardiovascular mortality.

Study or Subgroup	Single Coil Events	Single Coil Total	Dual Coil Events	Dual Coil Total	Weight	Odds Ratio IV, Random, 95% CI	Year
**2.2.1 RCT**							
Rinaldi 2003	1	38	1	38	2.6%	1.00 [0.06, 16.59]	2003
Bansch 2018	12	517	21	550	39.6%	0.60 [0.29, 1.23]	2018
**Subtotal (95% CI)**		**555**		**588**	**42.2%**	**0.62 [0.31, 1.24]**	
Total events	13		22				
Heterogeneity: Tau² = 0.00; Chi² = 0.12, df = 1 (P = 0.73); I² = 0%							
Test for overall effect: Z = 1.35 (P = 0.18)							
**2.2.2 N-RCT**							
Aoukar 2013	9	246	20	563	31.9%	1.03 [0.46, 2.30]	2013
**Subtotal (95% CI)**		**246**		**563**	**31.9%**	**1.03 [0.46, 2.30]**	
Total events	9		20				
Heterogeneity: Not applicable							
Test for overall effect: Z = 0.07 (P = 0.94)							
**2.2.3 Prospective Observational**							
Leshem 2017	6	357	28	2316	25.9%	1.40 [0.57, 3.40]	2017
**Subtotal (95% CI)**		**357**		**2316**	**25.9%**	**1.40 [0.57, 3.40]**	
Total events	6		28				
Heterogeneity: Not applicable							
Test for overall effect: Z = 0.74 (P = 0.46)							
**Total (95% CI)**		**1158**		**3467**	**100.0%**	**0.90 [0.57, 1.41]**	
Total events	28		70				
Heterogeneity: Tau² = 0.00; Chi² = 2.29, df = 3 (P = 0.51); I² = 0%							
Test for overall effect: Z = 0.46 (P = 0.64)							
Test for subgroup differences: Chi² = 2.17, df = 2 (P = 0.34), I² = 7.8%							

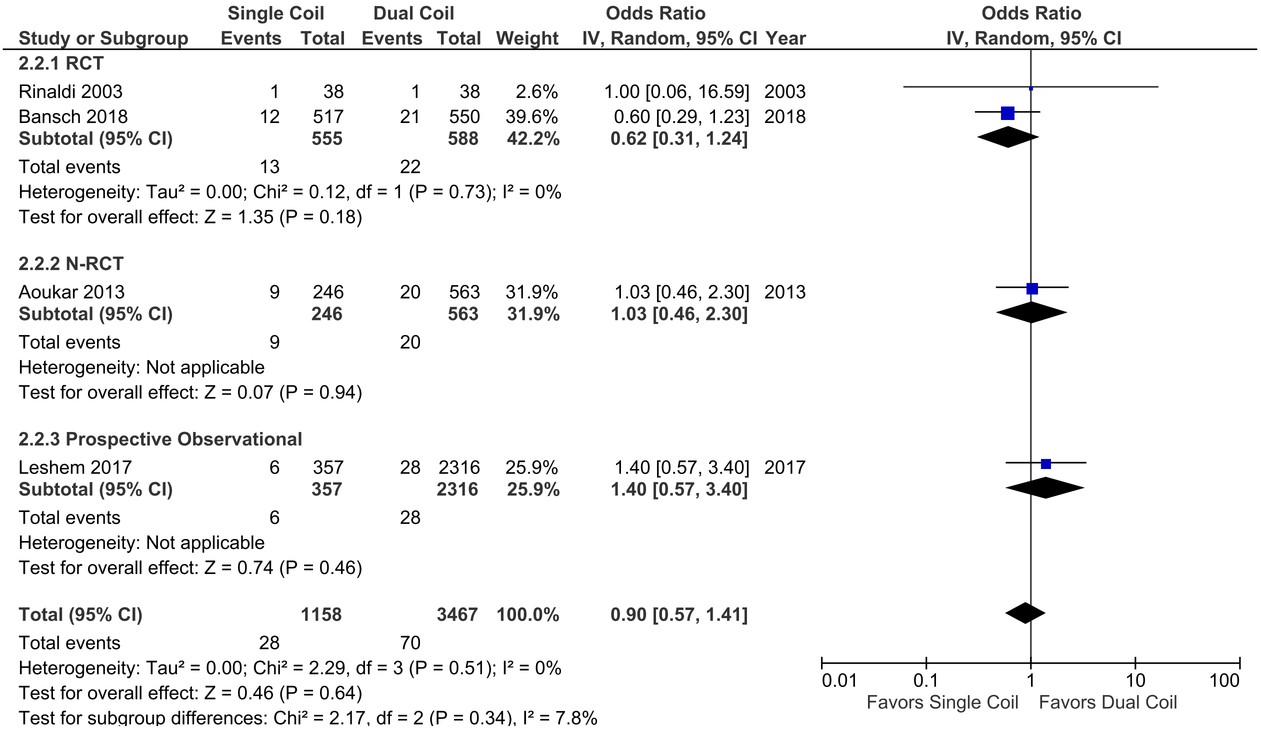

Supplementary Figure 9. Subgroup analysis on basis of study design for cardiovascular mortality.

Study or Subgroup	Single Coil Events	Single Coil Total	Dual Coil Events	Dual Coil Total	Weight	Odds Ratio IV, Random, 95% CI	Year
**2.2.1 More than 100**							
Aoukar 2013	9	246	20	563	31.9%	1.03 [0.46, 2.30]	2013
Leshem 2017	6	357	28	2316	25.9%	1.40 [0.57, 3.40]	2017
Bansch 2018	12	517	21	550	39.6%	0.60 [0.29, 1.23]	2018
**Subtotal (95% CI)**		**1120**		**3429**	**97.4%**	**0.90 [0.55, 1.48]**	
Total events	27		69				
Heterogeneity: Tau² = 0.02; Chi² = 2.28, df = 2 (P = 0.32); I² = 12%							
Test for overall effect: Z = 0.41 (P = 0.68)							
**2.2.2 Less than 100**							
Rinaldi 2003	1	38	1	38	2.6%	1.00 [0.06, 16.59]	2003
**Subtotal (95% CI)**		**38**		**38**	**2.6%**	**1.00 [0.06, 16.59]**	
Total events	1		1				
Heterogeneity: Not applicable							
Test for overall effect: Z = 0.00 (P = 1.00)							
**Total (95% CI)**		**1158**		**3467**	**100.0%**	**0.90 [0.57, 1.41]**	
Total events	28		70				
Heterogeneity: Tau² = 0.00; Chi² = 2.29, df = 3 (P = 0.51); I² = 0%							
Test for overall effect: Z = 0.46 (P = 0.64)							
Test for subgroup differences: Chi² = 0.00, df = 1 (P = 0.94), I² = 0%							

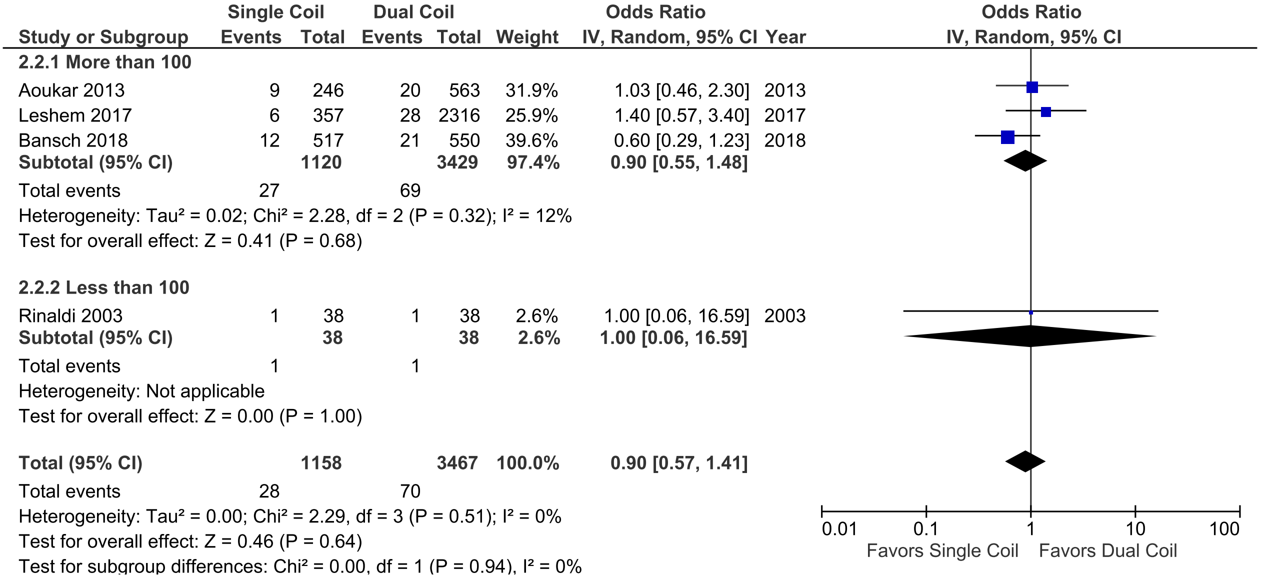

Supplementary Figure 10. Subgroup analysis on basis of number of patients for cardiovascular mortality.
